# Hospital and Institutional News

**Published:** 1919-09-06

**Authors:** 


### HOSPITAL AND INSTITUTIONAL NEWS.

### Charles Mercier, M.D., Lond., F.R.C.P., F.R.C.S.

IT is with deep sorrow that we have to announce the death of an old and treasured friend, Dr. Charles Mercier. We heard from him more than once during August, when he was cheered and rendered joyous by the surroundings of a new house he had just taken and the beautiful weather. Probably that month of August, despite his serious disabilities, was among the happiest of his life. On the 29th ultimo he was seized with severe abdominal pain and vomiting, followed by pleurisy and extreme weakness, and on Tuesday morning he passed peacefully away. Mercier was such a sufferer and so severely handicapped by illness—in the last few years failing sight and blindness being added to his troubles—that his maintenance of good spirits, continuous humour, and extraordinary working powers was indeed remarkable. He strove strenuously "to keep his friendships in repair," and had a multitude of friends who will miss him and treasure his memory. He was one of the kindest, ablest, and mentally strongest of men. To-day we must regretfully defer our tribute to his memory. *R.I.P.*

**The funeral will be on Friday, the 5th inst., at St. Peter's Church, Parkstone, at 2.30 p.m.**

### Prompt Action Taken.

THE leading article in THE HOSPITAL of the 30th ult., "Headquarters' Failure in India," on page 531, and the letter entitled "The Afghan Frontier: An Insufficient Medical Service," which we reprinted from the *Times*, on page 539, have been followed by prompt action on the part of the Indian Government and the Government at home. The letter gave a description of the Indian Government's mishandling of a grave position caused by the omission to extend just and considerate treatment to the British medical officers with temporary commissions who had volunteered for work in India during the war. The treatment that the Indian Government had extended to these members of the medical profession will probably go down to history as one of the most unwarrantable, cruel, and stupid policies ever adopted by a great Government. It was justly stated that the grave position thus caused in India could not fail to rouse the rightful condemnation of the British nation. This declaration seems to have gone home to those responsible for the abominations in question, for the *Times* has been able to publish the following notice in its issue of the 29th ult., some forty-eight hours after THE HOSPITAL, in which this criticism appeared, was circulated, and but seventy-two hours after the letter in question was published in the *Times* of the 26th August:—

136 Doctors for India: Preference for ex-Officers.

"The Secretary of State for India announces that 204 medical men are urgently required to fill vacancies in the Indian Medical Service. Of these two-thirds (136) will be Europeans and the remainder Indians. The appointment of European candidates will be made by nomination on the recommendation of a selection committee in England. Applications from Europeans, and from Indian candidates resident in this country, will be received at the India Office. Candidates must be over twenty-one and under thirty-two years of age at date of application. Preference will be given to candidates who are or have been serving with His Majesty's Forces during the war. All service rendered as a medical or combatant officer, or in a position usually filled by an officer, during the war will count both for promotion and pension on appointment to the service. The scale of pay has recently been greatly increased. A lieutenant on appointment now receives Rs.550 a month (equivalent at the present rate of exchange, the continuance of which cannot be guaranteed, to £605 per annum). Those who have had three years or more previous service will enter in the rank of captain on Rs. 700 a month (or £770 a year). An officer who is appointed to the service in 1919 or 1920 may claim to retire on a gratuity of £1,200 on completion of eight years' service from date of permanent appointment, provided he has given eighteen months' notice of his intention to retire. Application forms and further particulars can be obtained from the Secretary, Military Department, India Office. The correspondence should be clearly marked on the top left-hand side of the envelope, "Medical Recruitment."

### A Case for Immediate Explanation.

We should very much like to know, as the profession and public justly require to know, what has been done in the case of the wife of the middle-aged practitioner who bought a practice a few years before the war, increased it, married, and saved money, and, when the war came, joined for Indian service for a payment of 24s. a day. Since then the practice has dwindled away to nothing; his wife is in financial trouble at home, for now all his savings are spent, his child has died, his wife is ill and has had to undergo a severe operation, and his petitions for release and return have been met by the Indian Government with an official regret that only urgent cases can be considered. The India Office and the Indian Government has not hastened, as all impartial people were entitled to expect that they would, to state what steps have been taken to reverse the brutal decision regarding this practitioner and to find out, and provide with ample funds, the poor lady, our confrère's wife, who has suffered grievous injustice and hardship, owing to her husband's action in placing himself at the mercy of the Indian Government and the India Office. We must press, and we hope our confrères in the lay press will press, for an immediate explanation and assurance from Mr. Montagu, the Secretary of State for India, that this case has been dealt with promptly and with justice, by sending the middle-aged practitioner back to England and making full and ample compensation.

